# β-Caryophyllene, A Natural Dietary CB2 Receptor Selective Cannabinoid can be a Candidate to Target the Trinity of Infection, Immunity, and Inflammation in COVID-19

**DOI:** 10.3389/fphar.2021.590201

**Published:** 2021-05-14

**Authors:** Niraj Kumar Jha, Charu Sharma, Hebaallah Mamdouh Hashiesh, Seenipandi Arunachalam, MF Nagoor Meeran, Hayate Javed, Chandragouda R. Patil, Sameer N. Goyal, Shreesh Ojha

**Affiliations:** ^1^ Department of Biotechnology, School of Engineering & Technology (SET), Sharda University, Greater Noida, India; ^2^ Department of Internal Medicine, College of Medicine and Health Sciences, United Arab Emirates University, Al Ain, United Arab Emirates; ^3^ Department of Pharmacology and Therapeutics, College of Medicine and Health Sciences, United Arab Emirates University, Al Ain, United Arab Emirates; ^4^ Department of Anatomy, College of Medicine and Health Sciences, United Arab Emirates University, Al Ain, United Arab Emirates; ^5^ Department of Pharmacology, Delhi Pharmaceutical Sciences and Research University, New Delhi, India; ^6^ Shri Vile Parle Kelvani Mandal's Institute of Pharmacy, Dhule, India

**Keywords:** COVID-19, SARS-CoV-2, beta-caryophyllene, immunomodulators, natural products

## Abstract

Coronavirus disease (COVID-19), caused by novel severe acute respiratory syndrome coronavirus 2 (SARS-CoV-2), is an ongoing pandemic and presents a public health emergency. It has affected millions of people and continues to affect more, despite tremendous social preventive measures. Identifying candidate drugs for the prevention and treatment of COVID-19 is crucial. The pathogenesis and the complications with advanced infection mainly involve an immune-inflammatory cascade. Therefore, therapeutic strategy relies on suppressing infectivity and inflammation, along with immune modulation. One of the most promising therapeutic targets for the modulation of immune-inflammatory responses is the endocannabinoid system, particularly the activation of cannabinoid type 2 receptors (CB2R), a G-protein coupled receptor which mediates the anti-inflammatory properties by modulating numerous signaling pathways. To pharmacologically activate the CB2 receptors, a naturally occurring cannabinoid ligand, beta-caryophyllene (BCP), received attention due to its potent anti-inflammatory, antiviral, and immunomodulatory properties. BCP is recognized as a full selective functional agonist on CB2 receptors and produces therapeutic effects by activating CB2 and the nuclear receptors, peroxisome proliferator-activated receptors (PPARs). BCP is regarded as the first dietary cannabinoid with abundant presence across cannabis and non-cannabis plants, including spices and other edible plants. BCP showed tissue protective properties and favorably modulates numerous signaling pathways and inhibits inflammatory mediators, including cytokines, chemokines, adhesion molecules, prostanoids, and eicosanoids. Based on its pharmacological properties, molecular mechanisms, and the therapeutic potential of BCP as an immunomodulator, anti-inflammatory, organ-protective, and antiviral, we hypothesize that BCP could be a promising therapeutic and/or preventive candidate to target the triad of infection, immunity, and inflammation in COVID-19. In line with numerous studies that proposed the potential of cannabinoids in COVID-19, BCP may be a novel candidate compound for pharmaceutical and nutraceutical development due to its unique functional receptor selectivity, wide availability and accessibility, dietary bioavailability, nonpsychoactivity, and negligible toxicity along with druggable properties, including favorable pharmacokinetic and physicochemical properties. Based on reasonable pharmacological mechanisms and therapeutic properties, we speculate that BCP has potential to be investigated against COVID-19 and will inspire further preclinical and clinical studies.

## Introduction

COVID-19, a public health emergency and pandemic, has affected millions of people worldwide and continues to do so, despite numerous preventive measures, and this situation will continue until a vaccine is developed (Huang et al., 2020). The severity of infection varies from patients being asymptomatic to pre-symptomatic to symptomatic with different stages of illness, ranging from mild, moderate, to severe (Yang et al., 2020). The symptoms include fever, dry cough, sore throat, diarrhea, rashes on the skin, face, or toes, shortness of breath, loss of smell, anorexia, fatigue, headache, myalgia, anosmia, and ageusia, identified as the clinical criteria for diagnosis of COVID-19 (Jin et al., 2020). The majority of deaths are happening due to complications, such as severe pneumonia, acute respiratory distress syndrome (ARDS), shock, sepsis, and resultant multi-organ failure (Huang et al., 2020; Yang et al., 2020). The pathogenesis of COVID-19 emerges as a multifaceted, multi-system, multi-organ disorder, including viremia to overt the activation of immune responses and inflammatory processes that result in a dysregulated immune pattern, manifested by a massive rise in the levels of pro-inflammatory cytokines, chemokines, and adhesion molecules (Dhama et al., 2020). This causes the onset of a “cytokine storm” or “cytokine release syndrome”, which mainly causes ARDS and further leads to pathogenic effects through a quite ubiquitous target at a multiple-organ level (Dhama et al., 2020; Tang et al., 2020; Vinciguerra et al., 2020).

At present, many drugs are being repurposed for supportive management in COVID-19 based on docking studies, pharmacological rationale, and clinical experiences (Jean and Hsueh, 2020; Kandeel and Al-Nazawi, 2020; Rabaan et al., 2020; Singh et al., 2020). The pathogenesis and the complications developed with the infection mainly involve an immune-inflammatory cascade; therefore, the therapeutic strategies focus on reducing inflammation and immune modulation of this cascade (Dhama et al., 2020; García, 2020; Scavone et al., 2020; Zhou et al., 2020). Despite recent availability of vaccine for prophylaxis, massive efforts are ongoing for the discovery of novel drugs for the treatment and prevention of COVID-19 (Jean and Hsueh, 2020; Kandeel and Al-Nazawi, 2020; Rabaan et al., 2020; Singh et al., 2020). In parallel with repurposing modern medicines, there are numerous attempts to explore natural products with potential to target the interplay of viral infection and immune-inflammatory axis (Bahramsoltani and Rahimi, 2020; Basu et al., 2020; Benarba and Pandiella, 2020; Hensel et al., 2020; Mondal et al., 2020; Narkhede et al., 2020). Over the past few months it has been suggested that natural products hold great promise in the management of COVID-19 due to their antiviral, anti-inflammatory, and immunomodulator activities (Bahramsoltani and Rahimi, 2020; Basu et al., 2020; Benarba and Pandiella, 2020; Mondal et al., 2020; Narkhede et al., 2020). Thus, identifying candidate compounds which have selectivity against viral components as well as prevent viral entry, enhance immunity and attenuate inflammatory factors in host could be important in context to COVID-19.

Many propositions have been made on the possible therapeutic potential of essential oils-derived phytochemicals, including many terpenes or terpeno-alcoholic compounds, in COVID-19 (Asif et al., 2020; Boukhatem and Setzer, 2020; da Silva et al., 2020; Diniz et al., 2021). Many of the terpene components present in cannabis are widely consumed in food and used in traditional medicine (Anil et al., 2021). Some of these compounds showed potential to modulate the endocannabinoid system, which represents one of the newest therapeutic targets in regard to regulation of innate and adaptive immunity and immunomodulatory and anti-inflammatory properties. The endocannabinoid system is targeted by plant-derived compounds, termed phytocannabinoids, which have gained attention for therapeutic modulation of cannabinoid type-1 receptors (CB1R) and type-2 (CB2R), the components of endocannabinoid system (Oláh et al., 2017). The latest therapeutic strategy in targeting the endocannabinoid system is to activate the CB2R, a G-protein coupled receptor which appears to regulate immunity, inflammation, and pain. The activation of CB2R has been shown to exert potent anti-inflammatory, immunomodulatory, and organ-protective properties with no psychotropic effects, which are commonly observed with CB1R. Over the past few months, it has been suggested that modulation of the endocannabinoid system by cannabinoids, including cannabidiol, could be useful in prophylaxis and treatment of COVID-19 and may improve prognosis (Costiniuk and Jenabian, 2020; Esposito et al., 2020). Recently, extract of *Cannabis sativa* containing phytocannabinoids and terpenes were shown to modulate the inflammatory mediators in alveolar epithelial cells (A549) in COVID-19-associated inflammation and suggested that the phytocannabinoid mix formulation exerted better activity in comparison with individual fractions from cannabis (Anil et al., 2021). Many cannabinoids, including cannabidiol, have been suggested for their possible potential as preventive agents or therapeutic adjuvants with other agents in targeting the trinity of infection, inflammation, and immunity in COVID-19 (Byrareddy and Mohan, 2020; Costiniuk and Jenabian, 2020; Esposito et al., 2020; Nagarkatti et al., 2020; Sexton, 2020; Raj et al., 2021).

**FIGURE 1:**
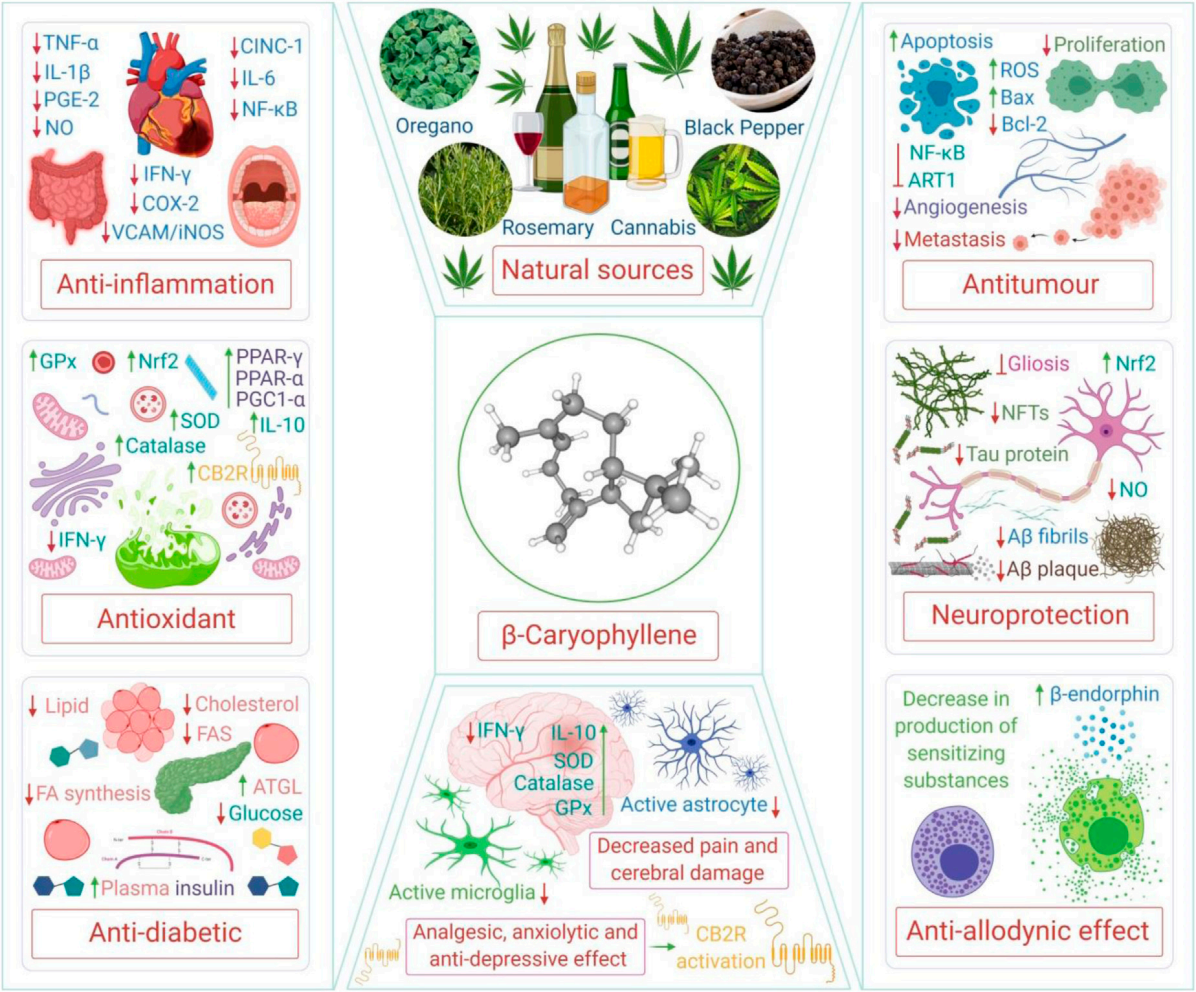
The structure and various polypharmacological properties and therapeutic potential of BCP. TNF-α, tumor necrosis factor alpha; IL, interleukin; PGE-2, prostaglandin E2; NO, nitric oxide; CINC-1, cytokine-induced neutrophil chemoattractant 1; NF-κB, nuclear factor kappa B; IFN-γ, interferon gamma; COX-2, cyclooxygenase-2; VCAM, vascular cell adhesion protein; iNOS, inducible nitric oxide synthase; GPx, glutathione peroxidase; SOD, superoxide dismutase; PPAR-γ, peroxisome proliferator-activated receptor gamma; PGC1-α, peroxisome proliferator-activated receptor gamma coactivator 1-alpha; Nrf2, nuclear factor erythroid 2–related factor 2; FAS, fatty acid synthase; ATGL, adipose triglyceride lipase; ROS, reactive oxygen species; Bax, Bcl-2 associated X protein; Bcl-2, B-cell lymphoma 2; ART1, arginine ADP-ribosyltransferase 1; NFTs, neurofibrillary tangles; Aβ, amyloid beta; CB2R, cannabinoid receptor type 2.

Among numerous cannabinoids, beta-caryophyllene (β-Caryophyllene; BCP), a naturally occurring terpene, has received enormous attention in the past few years due to its recognition as a full functional agonist on CB2R which imparts its therapeutic potential by mediating anti-inflammatory and immunomodulatory properties (Gertsch et al., 2008). BCP, chemically known as trans-(1R,9S)-8-Methylene-4,11,1 is the first dietary cannabinoid of natural origin, with an abundant presence in a variety of spice blends and citrus flavors, as an additive or preservative, and for aroma in food products and beverages (Gertsch, 2008; Gertsch et al., 2008). BCP is one of the constituents of commonly consumed edible plants, such as cinnamon (Cinnamomum spp.), basil (Ocimum spp.), pepper (Piper spp.), breakfast mint [*Perilla frutescens* (L.) Britton], coriander (*Coriandrum sativum* L.), chestnut (*Aesculus hippocastanum* L.), sage (*Salvia officinalis L.*), cubeb pepper (*Piper cubeba* L.f.), thyme (*Thymus vulgaris* L.), myrrh [*Myrrhis odorata* (L.) Scop.], curry leaves [*Murraya koenigii* (L.) Spreng.], hops (*Humulus lupulus* L.), cloves [*Syzygium aromaticum* (L.) Merr. & L.M. Perry], hemp (*Cannabis sativa* L.), lavender (*Lavandula angustifolia* Mill.), oregano (*Origanum vulgare* L.), and rosemary (*Rosmarinus officinalis* L.), among others. Recently, a majority of the plant derived compounds showed potential in COVID-19 due to their antioxidant and anti-inflammatory properties and are of limited occurrence in certain genera and species or to a specific individual plant that may limit supply and demand. However, BCP is unique in terms of wide dietary availability and accessibility across numerous plant genera and species (Sharma et al., 2016). Till date, the presence of BCP has been confirmed in more than two thousand plants, including edible, medicinal, and ornamental plants. BCP is mainly synthesized by plants as a defense mechanism against insects and aphids, and plays a role in pollination. It is usually localized in the aerial parts of the plants including leaves, flowers, spate, inflorescence, and buds, with a low presence in the stem, roots, and rhizomes (Sharma et al., 2016). The structure and various polypharmacological properties and therapeutic potential of BCP are depicted in Figure 1.

In a recent molecular docking study, 171 components, including BCP, present in the essential oils of numerous plants were analyzed against SARS-CoV-2 main protease (SARS-CoV-2 M^pro^), SARS-CoV-2 endoribonucleoase (SARS-CoV-2 Nsp15/NendoU), SARS-CoV-2 ADP-ribose-1″-phosphatase (SARS-CoV-2 ADRP), SARS-CoV-2 RNA-dependent RNA polymerase (SARS-CoV-2 RdRp), the binding domain of the SARS-CoV-2 spike protein (SARS-CoV-2 rS), and human angiotensin−converting enzyme (hACE2) (da Silva et al., 2020). Very recently in an *in silico* study, BCP was shown to target M^pro^ (3CL^pro^), the main protease in SARS-CoV-2 involved in the processing of translating the viral RNA into the viral polyproteins (Narkhede et al., 2020). BCP interacted with the amino acid residues of SARS-CoV-2 via pie-alkyl interactions and showed good affinity along with druggable properties (Narkhede et al., 2020). In another recent study, Muthuramalingam et al., 2020 (Muthuramalingam et al., 2020) carried out a cheminformatics and interactome study using *in silico* approaches and found that BCP is one of the potential compounds among 259 phytochemicals screened for targeting thirteen COVID-19 immune genes regulating numerous signaling pathways. The study unveiled that 154 compounds interact with COVID-19-associated immune genes. BCP and its derivative, β-caryophyllene oxide, was found to target immune genes, and was suggested useful for designing and developing as a potential agent against COVID-19 (Muthuramalingam et al., 2020).

**FIGURE 2:**
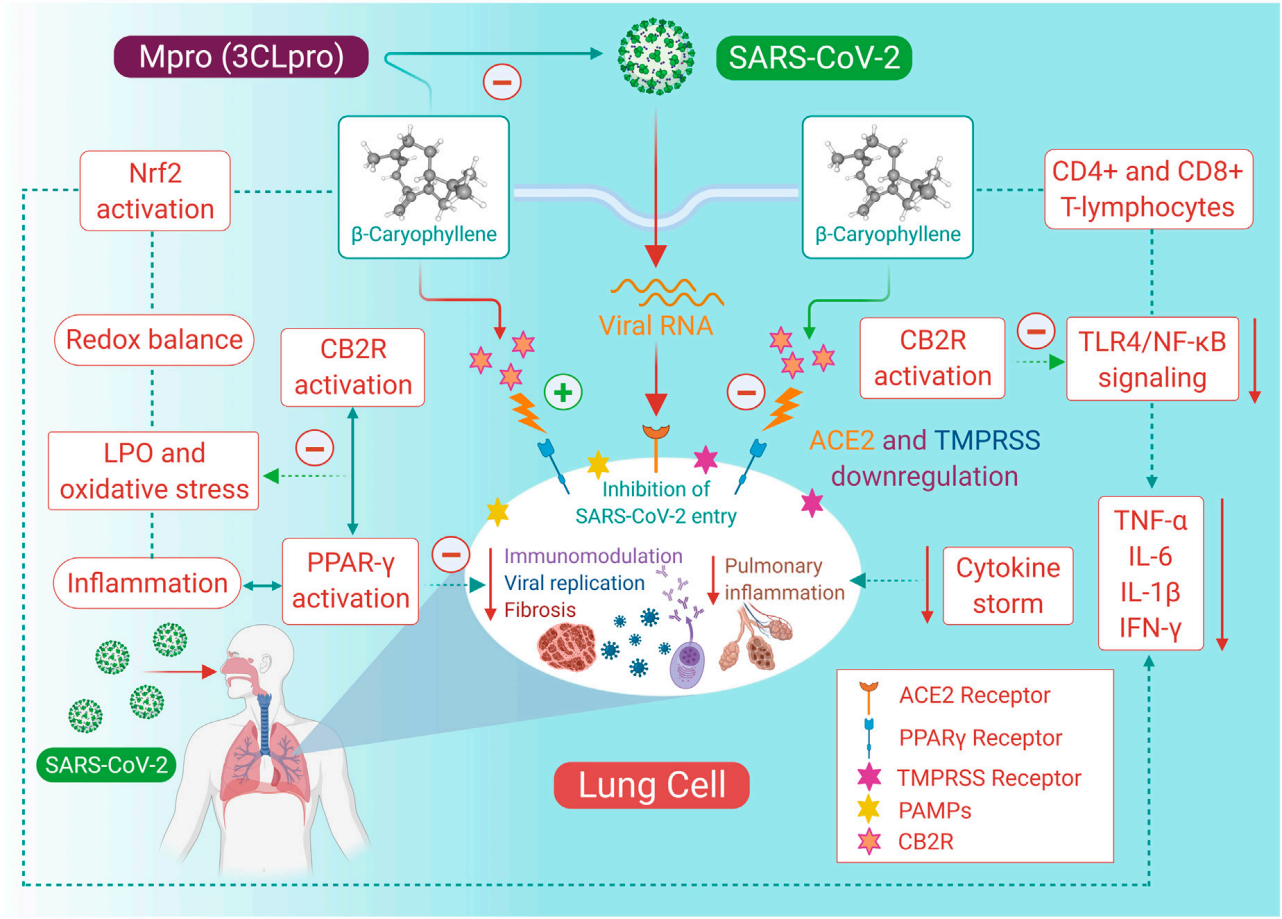
The proposed possible mechanisms and potential of BCP in COVID-19.

The present review scientifically contemplates the therapeutic prospects of BCP in COVID-19. The possibilities of BCP as a candidate in COVID-19 have been discussed based on reported findings, particularly immunomodulatory, anti-inflammatory, and antiviral properties. Additionally, CB2R activation has been suggested as a possible therapeutic target in COVID-19. Based on the role of CB2R in immune-inflammatory mechanisms, we hypothesized that BCP endowed with CB2R agonist properties may potentially limit the severity and progression of COVID-19 by modulating infection, immunity, and inflammation. The potent anti-inflammatory activity mediating multiple pathways and mediators of inflammation, including the inhibition of pro-inflammatory cytokines, chemokines, and adhesion molecules, along with the suppression of macrophage infiltration and neutrophil-endothelial cell interaction, might constitute a promising pharmacological and nutritional approach to inhibit the cytokine storm, which is a major reason for death in COVID-19. The potential of BCP in improving host cellular immunity against infection and its good antiviral and antibacterial activity, along with the antioxidant effects, may further help in controlling the symptoms and the worsening of the disease, secondary infections, complications, progression, and resultant death. BCP has potential to protect from the risk factors, prevent the entry of the virus, and ameliorate organ damage and the pathological manifestation of SARS-CoV-2 on the different organ systems. A scheme of the effect of BCP mediating CB2R activation has been proposed in context of infection, inflammation, and immunity in COVID-19 **(** Figure 2).

The literature reviewed herein indicates that BCP may be a promising candidate as a preventive and therapeutic agent or adjuvant for COVID-19 given its pharmacological and molecular mechanisms, including its CB2R agonist property, integrating with its antiviral, anti-inflammatory, and immunomodulatory properties in numerous experimental studies (Sharma et al., 2016). However, no study has yet directly demonstrated the efficacy of BCP against SARS-CoV-2 infections. But, based on pharmacological properties, a logical approach has been presented on the therapeutic potential of BCP in COVID-19.

## BCP as a Functional CB2 Receptor Agonist

Gertsch and colleagues first recognized BCP as a functional CB2R agonist using numerous model systems, including *in silico*, *in vitro*, and *in vivo* studies (Gertsch et al., 2008). In the molecular docking studies, BCP was observed to interact with CB2R on the same binding sites as that of CP55, 940, a CB2R agonist. It binds well in a hydrophobic sac involving lipophilic amino acid residues and it was suggested that the double bond with conformation E of BCP is vital for the receptor binding (Gertsch et al., 2008). Accumulating experimental studies have demonstrated the CB2R activation mediated effects of BCP in attenuating inflammation, oxidative stress, apoptosis, fibrosis, and immune modulation. The CB2R-dependent anti-inflammatory mechanism of BCP has been demonstrated in oral mucositis (Picciolo et al., 2020), glioblastoma (Irrera et al., 2020), neuropathic pain (Klauke et al., 2014; Aly et al., 2019), bipolar disorders (Hwang et al., 2020), wound healing (Koyama et al., 2019), interstitial cystitis (Berger et al., 2019), autoimmune encephalomyelitis/multiple sclerosis (Alberti et al., 2017; Askari et al., 2019), neurocognitive disorders (Lindsey et al., 2019; Chávez-Hurtado et al., 2020), arthritis (Irrera et al., 2019), metabolic and neurobehavioral alterations (Youssef et al., 2019), insulin resistance and vascular inflammation (Youssef et al., 2019), hyperglycemia (Basha and Sankaranarayanan, 2016), peripheral neuropathy (Segat et al., 2017), atherosclerosis (Zhang et al., 2017), cardiotoxicity (Meeran et al., 2019), osteoporosis (Shan et al., 2017), vascular dementia (Lou et al., 2017), dopaminergic neurodegeneration/Parkinson’s disease (Javed et al., 2016), Alzheimer’s disease (Cheng et al., 2014), cerebral ischemia-reperfusion (Poddighe et al., 2018), liver fibrosis (Mahmoud et al., 2014), pulmonary inflammation (Andrade-Silva et al., 2016), intestinal inflammation (Bento et al., 2011), acute myocardial infarction (Younis and Mohamed, 2019), acute renal injury (Horváth et al., 2012), diabetic nephropathy (Li et al., 2020), and lipid disorders (Youssef et al., 2019).

In the majority of the experimental models involving inflammatory states similar to those of human diseases, the principal pharmacological and molecular mechanism observed is the inhibition of pro-inflammatory cytokines, NF-κB, adhesion molecules, and chemokines and the subsequent modulation of signaling pathways, mainly involving toll-like receptors, opioid receptors, SIRT1/PGC-1α, AMPK/CREB, MAPK/ERK, Nrf2/Keap1/HO-1, and the activation of nuclear peroxisome proliferator-activated receptors (PPARs). Cannabinoids are known to interact or crosstalk with a family of PPARs, including three subtypes: PPAR-α, PPAR-β/δ, and PPAR-γ. These subtypes are encoded by distinct genes and are regulated by steroids and lipid metabolites and mainly control lipid and glucose homeostasis and inflammatory responses (O'Sullivan, 2016). PPAR-γ agonists, pharmacologically known as thiazolidinediones, are clinically available drugs for use as insulin sensitizers in insulin resistance/type 2 diabetes mellitus. Recently, thiazolidinedione has been suggested for repurposing in COVID-19 due to its potential to attenuate cytokine storms (Ciavarella et al., 2020). PPAR-γ agonists were shown to inhibit the replication of numerous viruses, including human immunodeficiency virus, respiratory syncytial virus, hepatitis B, and hepatitis C viruses (Skolnik et al., 2002; Du et al., 2017). Further, PPAR-γ agonists have been shown to reduce morbidity and mortality in influenza A virus infections (Bassaganya-Riera et al., 2010). The activation of PPAR-γ in resident alveolar macrophages was reported to significantly ameliorate pulmonary inflammation and enhance host recovery following respiratory viral infections (Huang et al., 2019). Following amelioration of the tissue damage, PPAR-γ activation also controls the overproduction of cytokines. Thus, BCP may pause the onset of the cytokine storm from resident macrophages.

In addition to activation of CB2R, BCP also activates PPAR-α which favorably modulates the lipid metabolism by increasing the ability of hormone nuclear receptors PPAR-α and estrogen-related receptor α (ERRα) to drive the transcription of fatty acid oxidation enzymes by increasing the levels of peroxisome proliferator-activated receptor-gamma coactivator 1α (PGC-1α), as well as stimulating sirtuin 1 (SIRT1) deacetylase activity (Zheng et al., 2013; Wu et al., 2014). The role of sirtuin in the transcription and replication of viruses is well known and the activation of PPAR-α and lipolysis showed to reduce hepatitis C virus genotype-associated lipid metabolic disorder in liver diseases (Patra et al., 2019). PPAR-α activation was also shown to beneficially influence inflammation in alveolar epithelial cells, suggesting a potentially beneficial role of PPAR-α in ARDS (Hecker et al., 2015). Thiazolidinediones have shown numerous adverse effects, such as weight gain, osteoporosis, heart failure, stroke, and an increased risk of urinary cancer. Since BCP is natural, non-toxic, and devoid of the adverse effects of synthetic cannabinoids, it could be a safer alternative over synthetics. Together, the role of BCP as a PPAR-γ, as well as a PPAR-α agonist, seems promising in the regulation of the lipid and glucose metabolism, along with additional regulatory roles on cell proliferation and differentiation, vascular homeostasis, and inflammation, and the immune systems. Thus, BCP may be possibly useful to control the orchestrated immune-inflammatory events in COVID-19.

## Immunomodulatory Properties of BCP

SARS-CoV-2 enters the host cells by binding to ACE2 receptors and the pathogen associated molecular patterns (PAMPs) on the virus alert innate immune cells, the anti-viral effectors, such as T CD8+ cells, NK cells, neutrophils, monocytes, and macrophages about the presence of the invading virus. The innate immune cells, which express pattern recognition receptors (PRRs), such as toll‐like receptors (TLRs), retinoic acid‐inducible gene I (RIG‐I)‐like receptors (RLRs), and nucleotide‐binding and oligomerization domain (NOD)‐like receptors (NLRs), detect PAMPs to achieve a suitable immune response against the invading pathogen (Keam et al., 2020). PRR and PAMP interaction triggers phagocytosis and stimulates the synthesis of pro-inflammatory cytokines, such as type I interferon, IFNα/β and type II, IFN‐γ, and chemokines, such as CXCL‐10 and CCL‐2, to onset an antiviral environment (Allegra et al., 2020).

In the case of severe infection, the viruses are sensed by monocytes, tissue macrophages, and resident dendritic cells, resulting in an uncontrolled pro-inflammatory cytokines (IFN, TNF‐α, IL‐1β, and IL‐6) production, leading to a phenomenon called a “cytokine storm”, which damages the respiratory epithelial cells of the host (Allegra et al., 2020). The immune responses are critical for the eradication of the virus and the resolution of the active disease. CB2R represents an important receptor target for immune regulation and is predominantly expressed by immune cells of the immune system, such as B cells, T cells, CD8+ lymphocytes, CD4+ lymphocytes, NK cells, neutrophils, macrophages, basophils, eosinophils, platelets, mast cells, dendritic cells, microglia, and astrocytes (Howlett and Abood, 2017). The CB2R are well expressed in several organs, including the liver, spleen, thymus, brain, lungs, kidneys, tonsils, nasal epithelium, and PBMC, which are present in the pancreas, uterus, and reproductive tissues (Cabral et al., 2015). Both cannabinoid receptors, CB1R and CB2R, play an important role in the modulation of the immune system, potentially inducing immunosuppression (Cabral et al., 2015; Hernández-Cervantes et al., 2017). The therapeutic targeting of CB2R has received enormous attention, since these novel therapeutic agents would have no psychotropic effects, as is the case with CB1R.

Human CB2R were first cloned in 1993 from the promyelocytic leukemia cell line HL-60 (Munro et al., 1993) and the first CB2R-deficient mouse was generated in 2000 (Buckley et al., 2000); therefore, CB2R represent the relatively newest therapeutic targets. Mice deficient in CB2R showed an increased susceptibility and vulnerability to influenza infection, demonstrating that CB2R are important in immunoregulation in respiratory viral infections (Kapellos et al., 2019). The activation of CB2R exerted potent immunomodulation, mediating cell death induction, cytokine suppression, and inhibition of cell proliferation, along with the stimulation of regulatory T cell induction and anti-inflammatory cytokines (Rieder et al., 2010; Karmaus et al., 2012). However, few studies notably demonstrate that CB2R may modulate susceptibility to the experimental cerebral malaria through a CCL17-dependent mechanism (Alferink et al., 2016).

Since the recognition of BCP as an agonist of CB2R, numerous studies have demonstrated the therapeutic benefits of BCP by suppressing immune-inflammatory cascade when CB2R are activated. The activation of CB2R was reported to suppress lung pathology in infants infected with acute respiratory syncytial virus by reducing the levels of cytokines and chemokines (Tahamtan et al., 2018). In HIV patients, the activation of CB2R was shown to impair a productive infection and viral transmission involving a crosstalk/interaction between CB2R (Costantino et al., 2012) and to inhibit the replication of the virus in monocytes and macrophages (Ramirez et al., 2013). Recently, BCP was shown to modulate systemic and local immunity in an experimental autoimmune encephalomyelitis model (Askari et al., 2019) and the immunomodulatory effect has been attributed to the ability of BCP to inhibit CD4+ and CD8+ T lymphocytes and pro-inflammatory cytokines (Alberti et al., 2017). The immunomodulatory activity of BCP was also explained by an enhanced phagocytic capability, following an increased lysosomal activity and nitric oxide production in macrophages (Carvalho et al., 2017).

Further, BCP exerted a potent immunomodulatory effect by simultaneously inhibiting both Th1 cytokines, including IL-2 and IFN-γ, and Th2 cytokines, including IL-4, IL-5, and IL-10, in primary splenocytes (Ku and Lin, 2013). Also, BCP is present in many plants, such as *Chrysanthemum indicum* L. (Hwang and Kim, 2013), *Pterodonem arginatus* Vogel (Alberti et al., 2014), *Myracrodruon urundeuva* Allemão (Carvalho et al., 2017), *Schizonepeta tenuifolia* (Benth.) Briq. (Ng et al., 2018), and copaiba oil (Urasaki et al., 2020), in which it exerts immunomodulatory activity. Taken together, the studies demonstrate that CB2R play a key role in balancing the immune response and BCP by activating CB2R, holding promise in the therapeutic modulation of immune-inflammatory changes in patients with SARS-CoV-2 infection.

**FIGURE 3:**
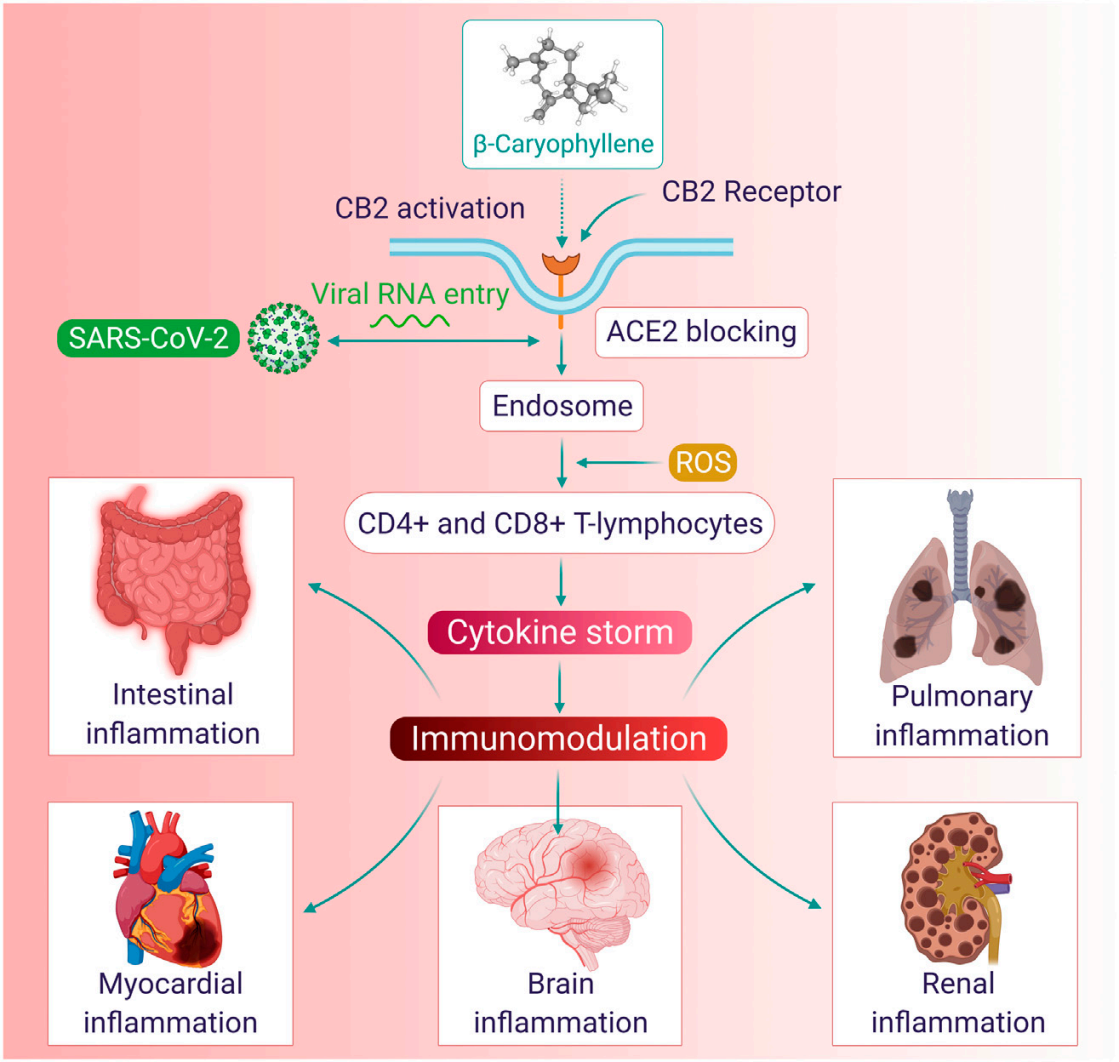
The immunomodulatory mechanisms and organ-protective effects of BCP.

In COVID-19, the use of immunomodulators is receiving attention and being regarded as a “sub-etiological treatment” in the absence of an effective antiviral drug. Additionally, BCP was shown to reduce ACE activity, which may be useful as ACE2 receptors, as the gateways of the virus entry, play a role from the entry of the virus to viremia and from respiratory distress to sepsis (Adefegha et al., 2017; Ajiboye et al., 2019). The immunomodulatory role of BCP is represented in Figure 3. Given the role of immunomodulators on the modulation of the hyperimmune-inflammatory response in COVID-19 patients, BCP may be a potential candidate for modulating immunity in patients at risk of infection and chronic metabolic and/or degenerative diseases, as well as in preventing the development and reducing the severity of COVID-19.

## Anti-inflammatory Properties of BCP

Cytokines regulate both inflammation and the immunopathology of viral infection. The massive production of proinflammatory cytokines is the key element that leads to an acute systemic hyperinflammatory state, to a cytokine storm syndrome, determining the intensity and severity of symptoms, eliciting the onset of acute respiratory distress, involving the extrapulmonary system, and increasing the risk of multiple organ failure and mortality during SARS-CoV-2 infection (Allegra et al., 2020). Mounting evidence demonstrates that BCP exerts potent anti-inflammatory properties in all body organs, including the liver, kidneys, brain, heart, pancreas, and blood, and suppresses systemic inflammation by inhibiting proinflammatory cytokines in macrophages and other inflammatory mediators, as well as signaling pathways (Yamaguchi and Levy, 2020). BCP was shown to exhibit a CB2R-dependent anti-inflammatory property by inhibiting lipopolysaccharide/endotoxin (LPS)-induced phosphorylation of kinases ERK1/2 and JNK1/2 in macrophages, since it is recognized as a CB2R agonist and a dietary cannabinoid (Gertsch et al., 2008). Macrophages in the lungs express CB2R, which, upon further activation by a CB2R agonist, reduced the release of pro-inflammatory cytokines (such as IL-6) and angiogenic factors (Staiano et al., 2016).

Many pathways were shown to be responsible for the anti-inflammatory activity in macrophages, including inhibiting the Ras-MAPK pathway, JNK pathway, TNF-α translation, and the inhibition of proinflammatory cytokines, including TNF-α (Gertsch et al., 2008; Rajesh et al., 2008). TNF-α triggers the activation of Ras, p38 MAPK, ERK1/2, SAPK/JNK, HMGB1/TLR4, and Akt pathways, and ultimately, the expression of proinflammatory cytokines, cellular proliferation, and migration (Gertsch et al., 2008; Rajesh et al., 2008). Additionally, numerous studies have also demonstrated the anti-inflammatory effects of BCP by activating CB2R and their subsequent pathways (Youssef et al., 2019). The CB2R-dependent anti-inflammatory effect of BCP has been demonstrated in inflammatory states of the heart (Meeran et al., 2019), liver (Cho et al., 2015; Arizuka et al., 2017; Varga et al., 2018), intestines (Cho et al., 2015), kidneys (Horváth et al., 2012; Hammad et al., 2018), lungs (Andrade-Silva et al., 2016), brain (Fontes et al., 2017; Yang et al., 2017; Askari et al., 2019; Askari and Shafiee-Nick, 2019), pancreas (Basha and Sankaranarayanan, 2016), urinary bladder (Berger et al., 2019), joints (Rufino et al., 2015; Irrera et al., 2019; D’Ascola et al., 2019), skin (Koyama et al., 2019), oral cavity, and blood (Brito et al., 2019). BCP also showed anti-inflammatory effects mediating histaminergic and arachidonic acid pathways (Oliveira-Tintino et al., 2018). Though, evidence supports that CB2R activation has anti-inflammatory effects, it has yet to be targeted to treat human disease.

BCP is present in many plants, such as *Campomanesia phaea* (O.Berg) Landrum (Lorençoni et al., 2020), *Pterodon pubescens* (Benth.) Benth. (Basting et al., 2019), *Ocimumm icranthum* Willd. (de Pinho et al., 2012), *Mosla dianthera* (Buch.-Ham. ex Roxb.) Maxim. (Wu et al., 2012), *Cordia verbenacea* A. DC. (Basting et al., 2019), *Duguetia furfuracea* (A.St.-Hil.) Saff. (Saldanha et al., 2019), *Cinnamomum osmophloeum* Kaneh. (Tung et al., 2008), *Croton campestris* A.St.-Hil., A. Juss. & Cambess. (Oliveira-Tintino et al., 2018), *Pinus spp.*(Basholli-Salihu et al., 2017 *)*, and *Copaiba oil* (Ames‐Sibin et al., 2018), and has been considered responsible for their anti-inflammatory effects by suppressing proinflammatory cytokines and other inflammatory mediators.

## Antiviral Properties of BCP

The role of plant-based natural products are well explored for their antiviral properties and are gaining attention for their therapeutic potential in COVID-19 (Mahmud et al., 2020; ul Qamar et al., 2020). The antiviral role of plant-derived compounds in inhibiting replication and blocking entry of viruses, including coronaviruses, in the host cells has been well reviewed elsewhere (Wen et al., 2007; Dhama et al., 2018; Hensel et al., 2020). The antiviral potential of many plant-derived compounds against SARS-CoV-2 has been recently demonstrated in *in silico*, *in vitro,* and *in vivo* studies (Bahramsoltani and Rahimi, 2020; Basu et al., 2020; Benarba and Pandiella, 2020; Mondal et al., 2020; ul Qamar et al., 2020). Many of the compounds showed targeting of SARS-CoV-2 using bioinformatic tools such as *in silico* analysis, molecular docking, or molecular farming to enhance the production of recombinant proteins including vaccines and antibodies (Rosales-Mendoza et al., 2020). In search of antiviral compounds, a library of plant-derived constituents containing 32,297 phytochemicals have been screened in molecular docking and results displayed that nine compounds, including myricitrin, methyl rosamarinate, licoleafol, and amaranthin, may curb the activity of 3CL^pro^ enzymes in SARS-CoV-2 (ul Qamar et al., 2020). The inhibitory activity on the proteases and other molecular targets should be assessed for specificity, affinity, dose-response, and kinetics in experimental studies. The binding of these compounds limits the availability of the substrate, modifies configuration of active sites, and prevents dimerization, viral entry, and/or viral replication. The role of cannabinoids against virus replication, maturation, transmission, and entry in particular has been demonstrated in *in silico* and *in vitro* studies (Khodadadi et al., 2020; Mohammed et al., 2020; Salles et al., 2020; Wang et al., 2020; Raj et al., 2021). It has become apparent that agents which have antiviral properties corroborated with anti-inflammatory and immunomodulatory properties are important to target the trinity of infection, inflammation, and immunity in context of COVID-19. To tackle SARS-CoV-2, the identification of viral protease appears as a striking therapeutic target to limit the replication of SARS-CoV-2 and many of the compounds are being investigated for their potential to target replication by inhibiting viral components such as M^pro^ (3CL^pro^), PL^pro^ and spike proteins. The protease of SARS-CoV-2 emerged as an attractive target to inhibit the replication of the virus. Recently, BCP was shown to target SARS-CoV-2 virus via pie-alkyl interactions to PHE 294 of SARS-CoV-2 with an affinity of -7.2 in an *in silico* docking study (Narkhede et al., 2020).

The antiviral properties of BCP or BCP-containing plants have been summarized in Table 1. The antiviral properties against herpes simplex virus type 1 (HSV-1) from the essential oils of many plants have been attributed to their chemical constituents, including BCP (Astani et al., 2011). The IC_50_ and TC_50_ for BCP were found to be 0.25 and 35 μg/ml, respectively (Astani et al., 2011). In plaque reduction assays, BCP exerted a concentration-dependent antiviral effect, with a selectivity index (ratio of TC_50_/IC_50_) of 140. BCP showed 98% reduction in infectivity, comparable to acyclovir, a standard antiviral drug. The authors suggested that BCP has potential to inactivate the herpes virus and may affect the structure of the virion envelope, which is essential for adsorption or entry into the host cells (Astani et al., 2011). BCP was shown to inhibit both Herpes Simplex Virus-2 (HSV-2) and acyclovir-resistant strain infections with a similar or lower selectivity index, compared to the BCP-rich essential oil of *Salvia desoleana* Atzei & V. Picci. However, BCP was not found to inhibit HSV-1 infection (Loizzo et al., 2008; Cagno et al., 2017). The selectivity index value >1 suggests that the compound has inhibitory action on viral replication and has low cytotoxicity on the host cells, thus a high selectivity index demonstrates better action of the compound. In the absence of guidelines on acceptability or appropriateness of selectivity index, values greater than 10 are considered better candidates for antiviral actions. The extracts rich in BCP displayed a very high selectivity index that indicates potent antiviral activity with negligible cytotoxicity on the host cells. The therapeutic efficacy and safety may also have different implications and should be taken in account considering the severity of viral infections and its onset, whether acute or chronic. BCP was tested in cell-based assays and showed a selectivity index of 71.1 in inhibiting the replication of the dengue virus (DENV-2). BCP acts as a viricidal by interfering with the very early steps of the viral replication cycle and *in silico* data showed that BCP specifically targets the dengue virus proteins. BCP was also found useful in Epstein-Barr virus-associated diseases. BCP has been recognized in the essential oil of *Waldheimia glabra* (Decne.) Regel, popularly known as ‘Ghaan-Poe’, is used for influenza in Tibetan medicine (Manzo et al., 2016; De et al., 2017). The essential oil showed antiviral activity against influenza virus H3N2 in an *in vitro* assay and was found comparable to ribavirin, a standard antiviral drug (Manzo et al., 2016).

**TABLE 1:** The antiviral activities of β-Caryophyllene (BCP) or BCP containing plants.

Sources	BCP (%)	Viral targets	References
*Mosla dianthera* (Buch. -Ham. ex Roxb.) Maxim	14.49	Influenza virus A (IVA)	Wu et al. (2012)
*Glechon spathulata* Benth	14.2	Human Herpes Virus Type 1 (HSV-1)	Venturi et al. (2015)
*Glechon marifolia* Benth	32.2	HSV-1	Venturi et al. (2015)
*Illicium verum* Hook.f	-	HSV-1	Astani et al. (2011)
*Buddleja cordobensis* Griseb	16.5	DENV-2, JUNV and HSV-1	Duschatzky et al. (2005)
*Cinnamomum zeylanicum* Blume	0.5–6.7	Influenza type A (H1N1)	Setzer (2016)
*Eupatorium patens* D. Don ex Hook. and Arn	14.1	HSV-1	García et al. (2003)
*Gaillardia megapotamica* (Spreng.) Baker	6.7	DENV-2, JUNV and HSV-1	Duschatzky et al. (2005)
*Hyptis mutabilis* (Rich.) Briq	10.9	Human Herpes Virus Type 2 (HSV-2)	Brand et al. (2015)
*Jungia polita* Griseb.	8.1	DENV-2, JUNV and HSV-1	Duschatzky et al. (2005)
*Lavandula angustifolia* Mill	5.1	H1N1	Setzer (2016)
*Lepechinia vulcanicola* J.R.I. Wood	8.7	HSV-1, HSV-2	Brand et al. (2015)
*Lippia turbinata* Griseb.	6.4	HSV-1	García et al. (2003)
*Melissa officinalis* L	14.2	HSV-2	Allahverdiyev et al. (2004)
*Ocimum campechianum* Mill	13.0	HSV-2	Brand et al. (2015)
*Thymus capitatus* (L.) Hoffmanns. and Link	2.9	Cytopathogenic murine norovirus	Moussaoui et al. (2013)
*Thymus vulgaris* L	7.0	HSV-1	Schnitzler et al. (2007)
*Zataria multiflora* Boiss.	3.0	Real time PCR (H9N2 subtype of AIV)	Shayeganmehr et al. (2018)

Further, the anti-inflammatory activity of essential oil was evidenced by the inhibition of NO production in LPS-stimulated macrophages and was found to be more potent than the standard drug dexamethasone. BCP was found in the essential oil of *Teucrium pseudochamaepitys* Georgi, an important Tunisian flora element that is used in traditional medicine for its antiviral activity against an enterovirus, Coxsackie 4 (CV-B4), known for causing myocarditis and CNS pathologies (Hammami et al., 2015). The essential oil showed potent antioxidant properties. The BCP-containing essential oil of *Glechon spathulata* Spreng. and *Glechon marifolia* Benth. are traditionally used in viral infections for their viricidal activity against HSV-1 strain KOS, VR733 (ATCC), or 29-R (ACV^res^) (Venturi et al., 2015). The essential oil of *Glechon spathulata* Spreng. exhibited activity against all strains and *Glrchon marifolia* Benth. was found to be active against two strains, KOS and VR733. HSV-1 was more susceptible to the oil of *Glechon spathulata* Spreng. than that of *Glechon marifolia* Benth. The viral titer was reduced by up to 2 log_10_ for KOS and VR-733 strains. BCP-containing essential oil of *Mosla dianthera* (Buch.-Ham. ex Roxb.) Maxim., a herb popularly used in respiratory illnesses, showed antiviral activity in mice infected with influenza virus A (Wu et al., 2012). It exerted potent antioxidant, anti-inflammatory, and antiviral effects, as evidenced by the reduced serum levels of IFN-γ and IL-4, viral titer in the lungs, amelioration of pneumonia, and an increased endogenous antioxidant level in the lung tissues. The findings were suggestive of its possible use in influenza and viral pneumonia (Wu et al., 2012). The essential oil obtained from *Fortunella margarita* (Lour.) Swingle, commonly known as Kumquats, which belongs to the citrus family, contained BCP and was shown effective against avian influenza-A virus (H5N1). BCP-containing essential oil of *Schizonepeta tenuifolia* (Benth.) Briq. was shown to inhibit norovirus replication through the induction of antiviral interferon production during virus replication by inducing the expression of both type I and type II interferons and increasing the transcription of interferon-β in infected RAW 264.7 cells via an increased phosphorylation of interferon regulatory factor 3, a critical transcription regulator for type I interferon production (Ng et al., 2018). Very recently, BCP on oral supplementation showed antiviral and immunomodulatory potential in an *in vivo* viral model of Newcastle disease virus (Hassanin et al., 2020).

In COVID-19 patients, the prevalence of coinfections has been reported and the co-pathogens may be bacteria, such as *Streptococcus pneumoniae, Staphylococcus aureus, Klebsiella pneumoniae, Mycoplasma pneumoniae, Chlamydia pneumonia, Legionella pneumophila*, and *Acinetobacter baumannii*, fungi, such as *Candida* species and *Aspergillus flavus*, or viruses, such as influenza, coronavirus, rhinovirus/enterovirus, parainfluenza, metapneumovirus, influenza B virus, and human immunodeficiency virus (Lai et al., 2020). Additionally, the antibacterial and antifungal effects of BCP have been reported in a number of studies (Schmidt et al., 2010; Rather et al., 2012; Dahham et al., 2015; Nieto-Bobadilla et al., 2015; Yang et al., 2015; Okoh et al., 2019;). Taken together, the antiviral and antibacterial activities, BCP may be a promising agent for secondary infections, as well as the viral infections.

## Antioxidant Properties of BCP

Besides the immune-inflammatory changes, macrophages and neutrophils can produce numerous reactive oxygen species (ROS), including H_2_O_2_, (O_2_^−^), (•OH), which further activates many signaling pathways and the onset of inflammation and cell death in many organs, including the lungs (Imai et al., 2008). Oxidative stress and the subsequent activation of NF-kB-toll-like receptor signaling pathways, triggered by viral pathogens such as SARS-CoV-2, are believed to amplify the host inflammatory response that results in acute lung injury (Saleh et al., 2020). Additionally, the hyper inflammatory/oxidative state may lead to the dysfunction of mitochondria, the hub of cellular oxidative homeostasis, and cause platelet damage, which, upon interaction with coagulation cascades, aggravates the clotting events and thrombus formation.

Mitochondrial oxidative stress may contribute to microbiota dysbiosis, altering the coagulation pathways and fueling the inflammatory/oxidative response, leading to a vicious cycle of events (Saleh et al., 2020). Oxidative stress further primes endothelial cells to acquire a pro-thrombotic and pro-inflammatory phenotype, predisposing patients to thromboembolic and vasculitic events and disseminated intravascular coagulopathy (Panfoli, 2020). Nrf2, a transcription factor which regulates the redox balance and the expression of genes involved in immunity and inflammation, is believed to defend against SARS-CoV-2 (McCord et al., 2020). The suppressed redox status of a cell enhances its susceptibility to oxidative stress, which may lead to cell death and viral release (Khomich et al., 2018). SARS-CoV-2 infections can lead to alterations of the redox balance in infected cells through the modulation of NAD^+^ biosynthesis and PARP function, along with altering the proteasome and mitochondrial function in cells, thereby leading to enhanced cell stress responses that further exacerbate inflammation. ROS production can increase IL-6 production and lipid peroxidation, resulting in cell damage (Nasi et al., 2020). Virus-induced inflammation and oxidative stress could be the common mechanisms responsible for the cardiovascular, pulmonary, renal, and neurological symptoms in COVID-19 patients (Nuzzo and Picone, 2020). BCP was found to exert protective effects in renal cells by suppressing ROS generation, NADPH oxidase 2/4 expression, and by controlling cell proliferation and inflammation by inhibiting proinflammatory cytokines, Nrf2/HO-1 and NF-κB/Nrf2 signaling pathways (Li et al., 2020).

BCP is present in *Ocimum sanctum* L. (Kamyab and Eshraghian, 2013), *Pinus spp.*(Xie et al., 2015 *)*, *Salvia officinalis* L. (El-Hosseiny et al., 2016), *Citrus limoni* (L.) Osbeck (Oboh et al., 2014), *Stachys pilifera *Benth. (Sadeghi et al., 2020), *Pistacia lentiscus* L. (Mohamed et al., 2018)*, Eplingiella fruticose* (Salzm. ex Benth.) Harley & J.F.B. Pastore (Beserra-Filho et al., 2019), *Lantana montevidensis* (Spreng.) Briq. (de Oliveira et al., 2019), *Azadirachta indica* A. Juss. (Okoh et al., 2019), *Rosmarinus officinalis* L. (Mohamed et al., 2016), *Aquilaria crassna* Pierre ex Lecomte (Dahham et al., 2015), and *Copaiaba oil* (Ames-Sibin et al., 2018) and has been shown to augment the levels of endogenous antioxidants, exerting ferric reducing properties, a Fe^2+^ chelation, and radicals scavenging activity in DPPH, FRAP, ORAC, ABTS, •OH, and NO assays (Oboh et al., 2014; Pant et al., 2014). BCP also enhances tolerance against stress, augments chaperons, and improves the antioxidant power (Srivastava et al., 2016). BCP mitigates the oxidative stress by counteracting ROS generation, inhibiting lipid peroxidation and glutathione depletion, free radical scavenging, and augmenting the endogenous antioxidant defense in the tissues of different organs, such as the heart (Ojha et al., 2016; Baldissera et al., 2017; Meeran et al., 2019), brain (Choi et al., 2013; Ojha et al., 2016; Tian et al., 2016), intestine (Bento et al., 2011), liver (Arizuka et al., 2017; Baldissera et al., 2017; Varga et al., 2018), stomach (Tambe et al., 1996), kidneys (Horváth et al., 2012; Hammad et al., 2018), pancreas (Basha and Sankaranarayanan, 2016), and blood (Youssef et al., 2019), which may aid the protective, as well as the adaptative, responses against viral infections and drugs.

BCP has been shown superior to probucol, α-humulene, α-tocopherol (Calleja et al., 2013), and synthetic CB2R agonist, JWH133 (Klauke et al., 2014). Also, BCP was shown to correct neurobehavior (anxiety, depression, and memory deficit), and neurochemical (oxidative, inflammatory, and neurotrophic factor) alterations in diet-induced obese rats (Youssef et al., 2019). Taken together, it is evident that BCP attenuated the oxidative stress and subsequent inflammation in organ dysfunction and metabolic disorders, favorably modulated redox signaling pathways (Baldissera et al., 2017; Varga et al., 2018), which are akin to the pathophysiology of SARS-CoV-2 infection.

## BCP may be Prospective in COVID-19 Associated Sepsis

SARS-CoV-2 infections may lead to sepsis and to subsequent multi-organ failure. Sepsis involves both the inflammatory response and immune suppression in response to an infection (Mira et al., 2017). CB2R plays a vital role in neutrophil/leukocyte recruitment, thereby suppressing infection and inflammation during sepsis (He et al., 2019). However, CB2R was also shown to contribute to septic immune dysfunction and mortality (Csóka et al., 2009). In a recent review the role of CB2R as a therapeutic target has been suggested based on the reports from preclinical animal models or *in vitro* cultured cells (He et al., 2019). The authors suggested that due to the lack of clinical evidence and the ambiguous underlying mechanisms, the clinical application of CB2R stimulation in sepsis is yet to be confirmed further (He et al., 2019). In many recent studies specific CB2R synthetic agonists, including HU-308 (Liu et al., 2020), GW405833 (Zhou et al., 2020), JWH133 (Çakır et al., 2020), and natural agonist, BCP (Brito et al., 2019), have been shown to ameliorate lung tissue damage, inhibiting oxidative stress, release of inflammatory mediators, recruitment of leucocytes and bacteremia, and improve survival in different preclinical models of sepsis. CB2R agonists were reported to ameliorate leukocyte adhesion to the endothelium, oxidative stress, systemic inflammatory mediators, microcirculatory dysfunction, bacteremia, and lung injury, along with an improvement in survival in experimental models of sepsis (Sardinha et al., 2014; Toguri et al., 2015). CB2R activation specifically mitigated septic lung injury by suppressing inflammatory mediators and augmenting autophagy (Liu et al., 2014; Liu et al., 2020). In an experimental model of polymicrobial sepsis, CB2R activation decreased the histopathological damage in the brain, heart, lungs, and liver by reducing the levels of caspase-3, p-NF-κB, TNF-α, IL-1β, and IL-6 in these tissues, as well as in the serum, and improved the anti-inflammatory cytokine IL-10 levels (Çakır et al., 2020).

To model sepsis, many of the experimental models rely on LPS-induced macrophages, which involve the activation and release of inflammatory mediators, including cytokines (Brito et al., 2019). BCP was reported to reduce the level of leukocytes, cytokines TNF-α, IL-6, IL-12, and IFN-γ, and increase the levels of IL-4 and IL-5 (Brito et al., 2019). BCP was shown to suppress inflammatory mediators and exert inhibitory effects on macrophages (Tung et al., 2008; Yamaguchi and Levy, 2019; Yamaguchi and Levy, 2020). Although the role of CB2R in sepsis has mixed reports, BCP has been shown to be beneficial in sepsis via CB2R activation and the off target effects cannot be excluded (Meza and Lehmann, 2018). Additionally, BCP is known to have a better safety profile over synthetic cannabinoids. Given the association of SARS-CoV-2 infections and sepsis-induced life-threatening organ dysfunction, BCP may be a promising candidate for COVID-19 associated sepsis.

## BCP may be Prospective in COVID-19 Associated Neurological Manifestations

SARS-CoV-2 is considered to be neurovirulent and neuroinvasive, in parallel with adherence to endothelial cells and cardiomyocytes (Sweid et al., 2020). Ischemic stroke, venous thrombosis, and intracerebral hemorrhage are the reported neurological manifestations of SARS-CoV-2 infection (Jiménez-Ruiz et al., 2020). The pathophysiology of ischemic stroke or cerebral hemorrhage includes an increased level of inflammatory cytokines in the brain, subsequent to the activation of microglia, astrocytes, and adhesion molecules, along with leukocyte recruitment and an impaired blood brain barrier. CB2R are upregulated during the inflammatory activation and CB2R agonists have been shown to be effective in acute ischemia and hemorrhagic stroke (Capettini et al., 2012).

BCP has been shown to exert a protective role on neurological deficit and neuroinflammation in experimental models, including middle cerebral artery occlusion induced-cerebral ischemia by suppressing the oxidative stress, inflammatory mediators, apoptosis, and reduction in brain edema, as well as preservation of tight junction proteins and repair of blood brain barrier (Zhang et al., 2017; Tian et al., 2019). BCP exerted its protective effects mediating CB2R activation (Choi et al., 2013) and its associated mechanisms, including the downregulation of TLR4 pathways to suppress inflammation and polarizing microglial phenotype from M1 to M2 (Tian et al., 2019), PI3K/Akt signaling pathway to suppress apoptosis (Zhang et al., 2017), an upregulation of the modulation of AMPK/CREB signaling (Choi et al., 2013), and the upregulation of Nrf2/HO-1 pathway to suppress oxidative stress and apoptosis (Lou et al., 2016).

BCP also attenuated neuronal necrosis, receptor-interaction protein kinase-1 (RIPK1), receptor-interaction protein kinase-3 (RIPK3) expression, and mixed lineage kinase domain-like protein (MLKL) phosphorylation in cerebral ischemia by inhibiting high-mobility group box 1 (HMGB1)-toll-like receptor 4 (TLR4) signaling pathways and proinflammatory cytokines. HMGB1, which is released by macrophages and monocytes in response to high levels of proinflammatory cytokines, plays a critical role in allowing innate immune cells to respond to both infection and injury. After its release, HMGB1 binds to its receptor for an advanced glycation of the end-products, which further activates MAPK and NF-κB, resulting in an overgeneration of various cytokines, causing a massive neutrophil infiltration into the lungs, and subsequent acute lung injury. The agents that target the release of HMGB1 are suggested to be useful in reducing mortality by preventing the progression from respiratory distress to sepsis (Wyganowska-Swiatkowska et al., 2020). Given the protective role of BCP on redox homeostasis and on the immune-inflammatory cascade in acute cerebrovascular disorders, it holds therapeutic promise for neurological manifestations of SARS-CoV-2.

## BCP may be Prospective in COVID-19 Associated Cardiovascular Conditions

SARS-CoV-2 infection has been reported to increase the susceptibility of patients affected by coronary artery disease and risk factors of atherosclerotic cardiovascular disease to develop adverse outcomes and lead to death (Vinciguerra et al., 2020). SARS-CoV-2 mediating ACE2 receptors infect endothelial cells, which regulate inflammation, vasomotor tone, and hemostatic balance. Pathological conditions associated with atherosclerotic progression, such as heart failure, coronary heart disease, hypertension, and diabetes mellitus, are the predictive factors for severity and susceptibility during SARS-CoV-2 infection (Vinciguerra et al., 2020). The pathogenesis involves endothelial dysfunction, altered vasopermeability, and formation of pulmonary microthrombi subsequent to inflammation, hypoxia, oxidative stress, mitochondrial dysfunction, and DNA damage. Patients with preexisting pulmonary vascular diseases also appear to have an increased risk of morbidity and mortality (Potus et al., 2020).

Atherosclerosis is considered as an ideal pathogenetic substrate for high viral replication ability, leading to adverse outcomes, as found in patients with cardiovascular factors. SARS-CoV-2 may aggravate atherosclerosis due to an excessive and aberrant plasmatic concentration of cytokines (Vinciguerra et al., 2020). Atherosclerosis involves vascular inflammation, characterized by a narrowed vascular lumen in the entire tunica intima and a reduced elasticity of the arterial walls. CB2R activation mitigated endothelial cell activation, transendothelial migration of monocytes, and monocyte/neutrophil-endothelial adhesion, and suppressed the proliferation and migration of human coronary vascular smooth muscle cells induced by TNF-α (Rajesh et al., 2007). A pneumonia causing pathogen, *Chlamydia pneumoniae*, provokes atheroma lesions by releasing heat shock proteins, which, by activating Hsp60 on endothelial cells, increase vascular smooth muscle cell proliferation. BCP was found to inhibit Hsp60-induced vascular smooth muscle cell proliferation and its potential in atherosclerosis has been suggested (Fukuoka et al., 2004). BCP also ameliorated acute myocardial injury by improving cardiac function, reducing infarct, restoring myocyte enzymes, and suppressing inflammation by inhibiting HSP-60/TLR/MyD88/NFκB signaling pathways (Younis and Mohamed, 2019). BCP was found to counteract drug-induced cardiomyopathy by attenuating inflammation, oxidative stress, and apoptosis by activating CB2R (Meeran et al., 2019). BCP mitigated hypercholesterolemia, dyslipidemia, and vascular inflammation, reduced atherogenic and coronary risk index, and corrected lipid metabolism by inhibiting proatherogenic vascular cell adhesion molecule 1 (VCAM-1) and restoring vascular eNOS/iNOS expression by maintaining the NO levels, mediating the activation of CB2 and PPAR-γ receptors in a high-fat diet and fructose-induced obesity (Baldissera et al., 2017; Harb et al., 2018; Youssef et al., 2019).

Furthermore, in addition to correcting the lipid profile, BCP-mediating CB2R-dependent mechanism inhibited leukocyte-endothelial attachment, neutrophil recruitment, and macrophage infiltration, inducing VCAM-1 to mediate the JAK2/STAT1/IRF-1 pathway (Zhang et al., 2017). BCP is one of the most important components of Copaiba oil, popularly used in Brazil for respiratory and cardiovascular illnesses. The nano-capsules of copaiba oil were shown to attenuate monocrotaline-induced pulmonary arterial hypertension in rats by counteracting the oxidative stress and inflammation, and by improving the cardiac function (Campos et al., 2017). One of the major clinical features and reasons for death in COVID-19 patients is respiratory distress syndrome, that also leads to acute cardiac injury (Huang et al., 2020). The potential of BCP on pulmonary vasculature is also promising and can be useful in reducing the risk of cardiopulmonary complications. The available studies are clearly suggestive of the therapeutic benefits of BCP in atherosclerosis, acute myocardial infarction, dyslipidemia, obesity, and fatty liver and could be important in preventing the worsening of the condition in COVID-19 patients.

## BCP may be Prospective in COVID-19 Associated Intestinal Inflammation

BCP was found to reduce the number of enterobacteria in the luminal and mucosal components, improving the clinical course of an intestinal inflammation in the mice model of colitis (Nieto-Bobadilla et al., 2015). BCP ameliorated intestinal inflammation in the animal models by mediating the activation of CB2 and the PPAR-γ pathway (Cho et al., 2007; Bento et al., 2011). It suppressed MPO activity and reduced the serum levels of protein and mRNA of IL-6 by 55% (Cho et al., 2007). IL-6 signaling pathway appears as one of the potential therapeutic targets for COVID-19. BCP also suppressed N-acetylglucosaminidase activity and the levels of mRNA expression of TNF-α, IL-1β, IFN-γ, chemokines, and the activation of extracellular signal-regulated kinase 1/2, NF-κB, IκB-kinase α/β, cAMP response element binding, and the expression of caspase-3 and Ki-67. BCP increased IL-4 levels and forkhead box P3 mRNA expression in the colon (Cho et al., 2007; Bento et al., 2011). In macrophages challenged with LPS, BCP reduced the levels of cytokines, such as TNF-α, keratinocyte-derived chemokines, and MIP-2.

Recently, in patients infected with SARS-CoV-2, an inflammatory response in the gut is evidenced by diarrhea and increased IL-6 and fecal calprotectin levels, showing the activation of neutrophils (Effenberger et al., 2020). Additionally, diarrhea appears as one of the most frequent symptoms in patients infected with SARS-CoV-2 (D'Amico et al., 2020). Given the role of BCP in suppressing intestinal inflammation (Cho et al., 2007; Bento et al., 2011) and diarrhea (Nieto-Bobadilla et al., 2015), BCP may hold great therapeutic promise for COVID-19.

## BCP may be Prospective in COVID-19 Associated Airway Inflammation

In many reports, vaccination of *Bacillus Calmette-Guérin* (BCG), a live attenuated vaccine of *Mycobacterium bovis* strain, is believed to provide protection against SARS-CoV-2 infection. BCG vaccination is believed to be associated with the induction of trained immunity, a kind of epigenetic reprogramming of innate immune cell types (Goodridge et al., 2016). In vaccinated individuals, monocytes and/or natural killer cells exhibit an upregulation of surface markers of activation and synthesis of cytokines, such as IL-1β, IL-6, IFN-γ, and TNF-α, in response to infection compared to non-vaccinated individuals; this helps in the faster clearance of pathogens, including influenza (Arts et al., 2018). BCP was found to ameliorate pulmonary inflammation in a mice model of *Mycobacterium Bovis* BCG-induced pulmonary inflammation by suppressing neutrophil accumulation, suppressing CXCL1/KC, LTB_4_, IL-12, and NO production, and mediating the CB2R activation (Andrade-Silva et al., 2016).

Additionally, BCP was also found to exert spasmolytic effects on the tracheal smooth muscle in the isolated organs (Pinho-da-Silva et al., 2012). BCP produced antispasmodic effects on the isolated tracheal smooth muscle of rats by inhibiting voltage-dependent L-type Ca^2+^ channels. BCP did not affect Ca^2+^ release from the intracellular storage. Further, the inhibitory effect on epithelial COX and a balance between relaxant and constrictor prostanoids exerted by BCP suggested that it may be useful in asthma-like conditions (Pinho-da-Silva et al., 2012). BCP containing essential oil of *Croton sonderianus* Müll. Arg. was found to exert myorelaxant activity in rat airway smooth muscles, which is suggestive of its potential in bronchospasm (Pinho-da-Silva et al., 2010). During viral infections, the activation of selective CB2R by agonists was shown to suppress leukocyte migration into the site of inflammation (Tahamtan et al., 2018). CB2 agonists in HIV-1 infection also reduced infection in primary CD4^+^ T cells, as well as CXCR4-activation-mediated G-protein activity and the phosphorylation of MAPK (Costantino et al., 2012). The CB2 selective property of BCP is reasonably speculated as a basis for its potential to inhibit virus replication, bacterial growth, and to regulate neutrophil recruitment, thus regulating inflammation.

The acute viral respiratory infections may increase the chances of secondary bacterial infections due to a compromised host immune response and thereby worsen the condition. SARS-CoV-2 was also reported to cause secondary bacterial infection (Dong et al., 2020). BCP is present in many plants, such as *Artemisia capillaris* Thunb. (Yang et al., 2015), *Juniperus rigida* var. hibernica Pshenn. (Meng et al., 2016), *Lavandula coronopifolia* Poir. (Ait Said et al., 2015), *Juglans regia* L. (Rather et al., 2012), *Mosla dianthera* (Buch.-Ham. Ex Roxb.) Maxim. (Wu et al., 2012), *Thymbra spicata* L. (Saidi et al., 2012), and *Lantana camara* subsp. *glandulosissima* (Hayek) R.W. Sanders (Tesch et al., 2011), which have been shown to exert inhibitory activity against respiratory pathogens and many virus, fungi, bacteria, and parasites in experimental studies and in human isolates.

## BCP may be Prospective in COVID-19 Associated Liver Dysfunction

Liver impairment has been reported in patients with SARS-CoV-2 infection (Feng et al., 2020; Sun et al., 2020) and it is believed to be due to systemic inflammation caused by a cytokine storm or pneumonia-associated hypoxia, and the drug regimens containing acetaminophen (Zhang et al., 2020). ACE2 receptors in the bile duct epithelial cells are expressed twenty times more than in hepatocytes and this plausibly explains that SARS-CoV-2 infection may cause bile duct epithelial cell damage (Lee et al., 2020). BCP was reported to ameliorate liver fibrosis in a bile duct ligation induced model, suppressing inflammation and apoptotic cell death by mediating the activation of CB2R (Mahmoud et al., 2014). BCP has also been reported to ameliorate drug induced liver injuries, such as ketoprofen-induced liver injury (Kelany and Abdallah, 2016), carbon tetrachloride-induced liver injury (Calleja et al., 2013), and D-galactosamine and lipopolysaccharide-induced liver failure by suppressing inflammation and mediating TLR4 and RAGE signaling pathways (Cho et al., 2015). BCP was partially attributed to the hepatoprotective effects of many plants, such as *Ocimum sanctum* L. (holy basil) (Kamyab and Eshraghian, 2013).

SARS-CoV-2 infection also increases vulnerability in patients with non-alcoholic fatty liver disease (NAFLD), a chronic liver disease characterized by hepatic steatosis (fatty liver), inflammation and hepatocyte damage (steatohepatitis), and lipotoxicity (Prins and Olinga, 2020). The expression of ACE2 is increased in cholangiocytes and hepatocytes during chronic liver damage and was increased in a diet-induced experimental model of NAFLD (Prins and Olinga, 2020). Metabolic perturbations, such as obesity, insulin resistance, hyperglycemia, dyslipidemia, and systemic hypertension, which constitute metabolic syndrome, are one of the risk factors of NAFLD (Friedman et al., 2018; Prins and Olinga, 2020). BCP showed a cholesterol-lowering effect by inhibiting the activity of hepatic hydroxy-methylglutaryl coenzyme A reductase in experimental models of hypercholesterolemia (Arizuka et al., 2017; Baldissera et al., 2017; Harb et al., 2018). Besides correcting the lipid metabolism, BCP also increased high density lipoprotein and attenuated liver injury and fibrosis, restored liver function enzymes and improved antioxidants (Harb et al., 2018).

BCP also attenuated chronic and binge alcohol-induced liver injury and inflammation by attenuating the pro-inflammatory phenotypic `M1` switch of Kupffer cells and by decreasing the expression of vascular adhesion molecules intercellular adhesion molecule 1, E-selectin, and P-selectin, as well as the neutrophil infiltration, and corrected hepatic metabolic dysregulation (Varga et al., 2018). BCP inhibited palmitate-inducible lipid accumulation in human HepG2 hepatocytes by activating AMPK mediating CB2R-dependent Ca^2+^ signaling pathway (Kamikubo et al., 2016). Mechanistically, BCP regulated hepatic lipid and glucose metabolism by modulating adenosine monophosphate (AMP)-activated protein kinase (AMPK), the main cellular energy sensor (Xu et al., 2018). Considering its hepatoprotective roles, BCP could be promising in conditions of liver injury associated with SARS-CoV-2 infection.

## BCP may be Prospective in COVID-19 Associated Renal Injuries

Acute kidney injury is one of the major complications in patients with SARS-CoV-2 infection (Cheng et al., 2020). ACE2 receptors located on the apical membrane and tubular cells facilitate viral entry and the infection elicits inflammatory responses that cause acute kidney injury (Fanelli et al., 2020; Soleimani, 2020). BCP ameliorated acute kidney injury in experimental models by attenuating renal impairment and tubular injury, suppressing renal inflammatory mediators, oxidative stress, apoptotic cell death, and preserving renal morphology via activation of CB2R (Horváth et al., 2012; Hammad et al., 2018).

BCP is present in many plants, such as *Stachys pilifera* Benth. (Sadeghi et al., 2020), *Salvia officinalis* L. (Koubaa et al., 2019), *Rosmarinus officinalis* L. (Mohamed et al., 2016), and *Pluchea indica* (L.) Less. (Sirichaiwetchakoon et al., 2020), and has been shown to be responsible for the renoprotective effects against drug induced-acute kidney injury, as well as diabetic and chronic kidney diseases, by restoring the renal function and suppressing oxidative stress, inflammation, and apoptosis. BCP also attenuated renal inflammation and oxidative stress by regulating NF-κB/Nrf2 signaling pathways in diabetic kidney diseases (Li et al., 2020). Given the increased risk of renal dysfunction in COVID-19 and the worsening of conditions in patients with chronic kidney or diabetic kidney disease, BCP may be a valuable candidate in preventing renal dysfunction in patients with COVID-19.

## Tissue Protective Effects of BCP may be Prospective in COVID-19 Associated Organ Injuries

Besides the lungs, the main site of virus entry and injury, SARS-CoV-2 infection may also affect other organs or organ systems, including the hepatic, renal, neurological, cardiovascular, musculoskeletal, gastrointestinal, hematological, olfactory, gustatory, ophthalmic, and cutaneous systems (Lai et al., 2020). Cardiac manifestations of SARS-CoV-2 involve endothelial damage, an altered lipid profile, endotoxemia, catecholamine, hypoperfusion, unstable hemodynamics, and drug-induced toxicity. BCP showed protective effects against catecholamine-induced myocardial injury and drug-induced cardiotoxicity by improving hemodynamics and alleviating endotoxemia by suppressing inflammation, oxidative stress, and apoptosis via activation of CB2R (Meeran et al., 2019).

The clinical manifestations of COVID-19 range from mild to severe with extensive involvement of the lungs, from pneumonia to ARDS, acute liver injury, acute cardiac injury, and neurological manifestations that may lead to multi-organ failure with a poor prognosis (Wang et al., 2020; Zhu et al., 2020). Severe lung disease with extensive alveolar damage and progressive respiratory failure leads to deadly outcomes (Yang et al., 2020). The fatalities are higher in older people with cardiometabolic diseases, cancer, immunocompromised patients, or patients with comorbidities. BCP was found to ameliorate renal dysfunction in acute and chronic kidney injury and diabetic kidneys.

BCP was found to be effective in liver failure by suppressing liver necrosis, fibrosis, and restoring liver function, mediating CB2R activation. BCP has been shown to be neuroprotective in models of cerebral ischemia, dopaminergic neurodegeneration, seizures, dementia, neurocognitive disorders, depression, anxiety, and encephalitis. BCP improved systemic inflammation and oxidative status with no hepatotoxicity, as with nonsteroidal anti-inflammatory drugs (Ames‐Sibin et al., 2018). It also reduced nausea, epigastric pain, and diarrhea, and improved gastrointestinal activity (Patra et al., 2010). BCP was also found to promote wound healing by modulating numerous signaling pathways (Parisotto-Peterle et al., 2020). Hematological abnormalities, including lymphopenia and leukopenia, have been reported in COVID-19 patients (Ding et al., 2020). The occurrence of leukopenia induced by chemotherapeutic drugs in an experimental model has been shown to be prevented by BCP (Campos et al., 2015).

Upon oral administration, BCP was found to be bioavailable in almost every organ, including the liver, kidneys, heart, lungs, and blood (Pant et al., 2019). BCP was shown to modulate stress-related genes, provide resistance against stress, improve life span, reduce ageing, and was considered one of the best adaptogenic compounds to enhance the tolerance against stress. The interactions between phytocannabinoids and terpenoids have been suggested to exert synergy for the therapeutic benefits in pain, inflammation, depression, anxiety, addiction, epilepsy, cancer, and microbial infections (Russo, 2011). Given the impact of COVID-19 on organ functions and considering the organ-protective effect of BCP, it is reasonable to hypothesize that the organ-protective activity of BCP will be beneficial in COVID-19.

## Safety and Toxicity of BCP

The United States Food and Drug Administration (USFDA) included BCP in the list of compounds regarded as Generally Recognized as Safe (GRAS) for its use as an additive and preservative in food products and beverages. BCP was shown to modulate the expression of drug metabolizing enzymes (phase I and II) in cell lines, rodents, and human liver microsomes, which may influence the bioavailability and efficacy of concomitantly administered drugs (Ambrož et al., 2019). BCP was shown to have a chemopreventive effect and is free from genotoxicity (Álvarez-González et al., 2014), mutagenicity (Di Giacomo et al., 2016), and clastogenicity (Di Sotto et al., 2010).

BCP exerted synergistic and/or additive actions with many drugs including azithromycin (Zhang et al., 2020), atovaquone (Zhang et al., 2020), metaxolone (Yamaguchi and Levy, 2020), imipramine (Askari et al., 2019), fluoxetine (Askari and Shafiee-Nick, 2019), docosahexaenoic acid (Brito et al., 2019), curcumin (Srivastava et al., 2016; D’Ascola et al., 2019), baicalein (Yamaguchi and Levy, 2016), catechin (Yamaguchi and Levy, 2016) and vitamins, which are suggested to be useful for repurposing for COVID-19. In many experimental studies, BCP was found to be better than the standard modern drugs such as phenylbutazone (Basile et al., 1988), probucol (Calleja et al., 2013), tocopherol (Calleja et al., 2013), ribavirin (Wu et al., 2012), atorvastatin (Campos et al., 2015), glibenclamide (Basha and Sankaranarayanan, 2016), and pioglitazone (Youssef et al., 2019). BCP delivered by inhalation was found to be bioavailable in the saliva and appears safe and tolerable (Tarumi and Shinohara, 2020). BCP was convincingly shown to mitigate drugs or xenobiotics-induced organ injuries; for example, it was found to improve the therapeutic efficacy of immunosuppressive drugs and reduce their side effects, such as myelosuppression and hepatotoxicity in experimental arthritis (El-Sheikh et al., 2019). BCP studied at the therapeutic doses was found devoid of organ toxicity in the experimental studies.

## Clinical Efficacy and Safety of BCP

BCP administered orally at a dose of 126 mg/day was evaluated in patients with peptic ulcer in a randomized double-blind, placebo-controlled trial (Shim et al., 2019). BCP improved dyspepsia symptoms by reducing *Helicobacter pylori* infections, improving nausea and epigastric pain, and mediating the inhibition of proinflammatory cytokines (Shim et al., 2019). BCP (3%) was evaluated in nineteen women for 20 min using an odor exposure device and was found to improve the libido and vaginal sensation during intercourse in women by improving the salivary testosterone concentrations with no effect on estrogen (Tarumi and Shinohara, 2020).

BCP was administered to diabetes patients with diabetes-related complications; painful distal symmetric polyneuropathy was found to relieve polyneuropathy with an increased amplitude and a reduction of pain, with good tolerance and no adverse effects (Semprini et al., 2018). Recently, in a placebo-controlled clinical study, patients with hand arthritis applied BCP-containing copaiba oil topically and BCP was found to be safe, well tolerated, and beneficial in reducing pain and inflammation (Bahr et al., 2018).

## Dosage Forms and Pharmaceutical Development of BCP

Many formulations containing BCP have been developed, including Amukkara Choornam (Patra et al., 2010), CIN-102, a coated pellets and matrix mini-tablet (Nieto-Bobadilla et al., 2015), and PipeNig®-FL, a high standardized content of BCP (Geddo et al., 2019). BCP is highly lipophilic, less soluble in water and, upon exposure to air, it easily oxidizes. To overcome its low bioavailability, many novel drug delivery systems have been developed. Various kinds of formulations, such as liposomes, nanoemulsions, nanofibers, microemulsions, nanoparticles, micelles, phospholipid complexes, nanocarriers, nanocomposites, hydrogels, and matrix formulations using cyclodextrin, have been developed to enhance the solubility, stability, and release pattern of BCP (Santos et al., 2018). Novel formulations will pave the way for the pharmaceutical development of BCP and may aid in improving its clinical usage.

## Limitations

Since the emergence of COVID-19, a significant number of natural products, including plant extracts and phytochemicals, have been proposed for their possible use as a preventive agent or as an adjuvant in COVID-19 (Asif et al., 2020; Boukhatem and Setzer, 2020; da Silva et al., 2020; Diniz et al., 2021). Though, given their pleiotropic and immunomodulatory nature, the role of phytocannabinoids are reasonably suggested useful, but caution should be exercised. The potential application of cannabinoids in COVID-19 management can’t be overlooked until proof-of-concept studies become available (Pastor et al., 2020; Anil et al., 2021). The cannabinoids shown to possess potent immunomodulatory, anti-inflammatory, and antimicrobial properties are proposed for their use in COVID-19. However, few of the phytocannabinoids have been screened in molecular docking studies for their potential activity against viral targets using the *in-silico* tools. The role of phytocannabinoids is believed to be delicate given their action on inflammation and immune modulation and the possibility of the unfavorable effects in acute infection due to risk of immunosuppression (Sexton, 2020). Among numerous cannabinoids, BCP has been shown to be more convincing in terms of its immunomodulatory, anti-inflammatory, and antiviral effects. BCP is one of the main compounds identified in a large number of dietary plants and is widely accessible and well-studied for its therapeutic benefits. However, the safety and efficacy of BCP still needs to be established in preclinical and clinical trials for its evidence-based use and application in humans. BCP is a functional agonist of CB2R and devoid of psychotropic effects, which makes BCP a fascinating candidate molecule for further investigation.

A majority of the experimental research carried out on the therapeutic benefits of phytochemicals are based on ethnopharmacological usage of the particular plant rich in these. Many have also been evaluated for their antiviral properties in addition to their anti-inflammatory and immunoregulatory roles in numerous immune-related disease models. Since the emergence of COVID-19, the repurposing of drugs began first with target identification and continues to be used in the screening of druggable agents against viral infections. It is noteworthy to state that until recent years, the antiviral potential of natural products were shown to be effective in *in vitro* studies, whereas the SARS-CoV emerged in 2003 (Kim et al., 2014; Park et al., 2017). But none of them have been evaluated meticulously enough to translate their effects to humans despite their potential efficacy in preclinical studies. This is due to many reasons, including the lack of an integrated approach. A recent report suggested that if an integrated and rigorous approach could have been followed since the emergence of SARS-CoV, we may have progressed to clinical studies and developed some useful agents in the process of drug discovery and development, which involves the testing of druggable compounds from laboratory to clinics (Pandey et al., 2020). It can be proposed that the phytochemicals should be investigated and validated in the preclinical models of COVID-19 despite strong evidence for their anti-inflammatory, immunomodulatory, and antiviral properties.

In the present manuscript, the possible role of BCP in COVID-19 has been proposed based on the previously reported potent pharmacological activity of BCP against infection, inflammation, and immunity in experimental models of human diseases other than SARS-CoV-2. Many authors proposed the hypotheses that CB2R, an important constituent in endocannabinoid system, may play a role in targeting the trinity of infection, inflammation, and immune dysregulation (Nagoor Meeran et al., 2020). Given the role of CB2R activation in attenuating inflammation, viral replication, and favorable modulation of immune systems, BCP endowed with the CB2R selective agonist property has been pharmacologically reasoned to be a candidate for its possible use as preventive agent or therapeutic adjunct in COVID-19. There are reports of long-term complications in some patients even after recovery from COVID-19. Thus, given the tissue protective effects and effect on numerous tissues remodeling effects, BCP could be a candidate to be investigated for possible use in improving prognosis and combating the long-term complications in COVID-19. Taking into consideration the safety of BCP in humans, dietary use, and efficacy of BCP in various disease models in experimental studies, BCP may be a valuable agent to be investigated further for COVID-19.

The available reports clearly demonstrate that the progression and complications of COVID-19 involves cytokine storm, therefore, cannabinoids activating CB2R may inhibit cytokine storm due to their additional organ-protective effects. However, until now there has been no clear evidence available on the antiviral activity of BCP on SARS-CoV-2. There is no preclinical or clinical data available on whether BCP can protect against COVID-19 or may be useful in treatment of COVID-19. The recent availability of animal models could be important in evaluating its preclinical efficacy. There is a lack of clinical data and rigorous pharmacokinetics in humans. Therefore, preclinical evaluation, including duration of use and dose, is suggested. The safety and interaction with concomitant drugs, as well as the heterogeneity of the target population, should also be considered before the possible use of BCP whether in prevention or as adjunct treatment. Nonetheless, there is an opportunity for further investigation to investigate the possible use against COVID-19. Considering the safety evidenced in numerous experimental and few clinical studies, further studies are encouraged to recommend the clinical usage and pharmaceutical development of BCP.

## Conclusion

BCP is a unique molecule in various ways, such as being dietary, devoid of psychotropic effects, possessing negligible toxicity, wide availability in plants, oral bioavailability, a druggable property, and functional receptor selectivity. BCP interacts or binds to different receptors, including CB2, PPAR-α, and PPAR-γ, opioid, histaminergic, TRPV, and TLR, and has enzyme inhibitory activities, including amylase, lipase, α-glucosidase, HMG-CoA reductase, acetylcholinesterase, secretase, cyclooxygenase, and nitric oxide synthase. Taken together, the receptor selectivity made it a distinctive candidate with a pharmacological rationale for pharmaceutical development, more than an antioxidant molecule, which is common with natural products-based nutraceuticals. Integrating the potent anti-inflammatory, immunomodulator, and antiviral properties of BCP and its potential benefits in pathological features of cardiovascular, neurological, gastrointestinal, hematological, renal, ocular, and cutaneous systems, which are the common accompaniments of SARS-CoV-2 infection, the benefits of BCP and plants rich in BCP may be important for COVID-19. The candidature of BCP in COVID-19 treatment may appear somewhat speculative but cannot be overlooked as it possesses favorable physiochemical and druggable properties with dietary use. However, the suggestion on the possible use of BCP in COVID-19 remains inconclusive until the *in-silico* observations could be confirmed in the experimental studies and further proof of the concept studies.

The polypharmacological properties, including receptor selectivity provide rationale and its drug-like properties, provide more realism for its future in drug discovery and development. Additionally, the antioxidant, anti-stress, and longevity potential provide a nutritional basis of its use to boost the immunity and suppress overt oxidative stress and subsequent hyperinflammatory states. Considering the recognition of safety status by USFDA and its favorable pharmacokinetic and physicochemical properties, BCP itself or plants containing a high amount of BCP may be important for nutritional or dietary usage. BCP and the plants containing BCP as a major ingredient may be candidates for developing novel antiviral and immunomodulator therapies for coronaviruses. However, further research is needed to address novel drug discovery employing the chemical scaffold or pharmacophores of BCP. The natural dietary availability and CB2 receptors mediated functional properties and selectivity are suggestive of developing BCP-based nutraceuticals and pharmaceuticals as candidate compounds for COVID-19 and other coronavirus diseases. The opinion of the authors on possible candidature for possible use of BCP in COVID-19 is solely based on available literature on the effects of BCP against infection, immunity, and inflammation in corroboration with the *in-silico* studies. The authors do not promote the use of BCP in any form for COVID-19 until clear evidence becomes available from proof of the concept studies.
